# Effect of Ultrasonic Pretreatment on the Far-Infrared Drying Process and Quality Characteristics of Licorice

**DOI:** 10.3390/foods12122414

**Published:** 2023-06-19

**Authors:** Jianwei Shang, Qian Zhang, Tongxun Wang, Yanrui Xu, Zepeng Zang, Fangxin Wan, Yuanman Yue, Xiaopeng Huang

**Affiliations:** College of Mechanical and Electronical Engineering, Gansu Agricultural University, Lanzhou 730070, Chinazangzp@st.gsau.edu.cn (Z.Z.);

**Keywords:** licorice, far-infrared drying, ultrasonic pretreatment, drying kinetics, quality

## Abstract

In this paper, the effects of different ultrasonic pretreatment processes on the far-infrared drying characteristics, quality indexes, and microstructure of licorice are evaluated. The results showed that ultrasonic pretreatment, combined with far-infrared drying, significantly reduced the drying time and moisture content of licorice compared with those of the control group. The highest total flavonoid content was obtained at an ultrasound power of 80 W. The total phenolic content (0.686 mg gallic acid equivalent/g) was higher than that in the control group, the increase was 19.4%, and its content was the highest at the sonication frequency of 20 kHz. The antioxidant capacity tended to increase and then decrease with the increase in sonication time, sonication power, and sonication frequency, and was the highest at 30 min of sonication. The soluble sugar content (31.490 mg glucose equivalent/g) was the highest at 30 kHz and 30 min. Observation of the microstructure revealed that the surface structure of the ultrasonic pretreated licorice slices changed significantly, forming more micropore channels, which facilitated the mass heat transfer during the drying process. In conclusion, ultrasonic pretreatment can significantly improve the quality of licorice tablets and significantly reduce the time required for subsequent drying. The combination of pretreatment parameters of 60 W ultrasonic power and 40 kHz ultrasonic frequency for 30 min was found to be an optimal combination of pretreatment parameters; therefore, this study may provide a technical reference for the industrialization of licorice drying.

## 1. Introduction

Licorice is the dried root of *Glycyrrhiza uralensis* Frich, *Glycyrrhiza inflate* Bat, or *Glycyrrhiza glabra* L. [1]. Licorice is internationally popular as a medicinal plant due to its high value multipurpose medicinal components, including triterpene glycyrrhizic acid and the flavonoids liquiritin, isoliquiritin, liquiritigenin, and osoliquiritigenin [2,3], which have anti-allergic, anti-viral, and immunomodulatory pharmacological effects and are often used in the treatment of coughs, oral diseases, and liver damage [4]. Licorice boasts high medicinal and economic value. However, due to the large water content of fresh licorice, it is prone to mildew and corruption during long-term storage and transportation, which reduces its edible value. As an important food preservation technology, drying can effectively reduce the activity of water in food and inhibit the growth of microorganisms, thus reducing its storage time at room temperature.

The traditional drying method is sun drying, but the drying time is long and subject to the weather, which is not conducive to the retention of the medicinal components of heat-sensitive medicinal plants. In recent years, some scholars have applied hot air drying, vacuum drying, microwave drying, vacuum freeze drying, and other methods to licorice to extend its storage period and reduce the loss of active ingredients [4,5,6]. Icier et al. studied the drying characteristics, energy/combustion efficiency, and quality properties (color and the total phenolic content) of licorice samples subjected to infrared drying (ID) and carbon fiber assisted drying (CFACD). The results show that the drying time of ID is shorter, but the CFACD method is better for maintaining color characteristics and improving energy/combustion efficiency [7].

The energy released by the infrared radiation source penetrates the surface of the material and can simultaneously heat the surface and interior of the material to achieve uniform internal heating and rapid dehydration [8]. In the study of kiwifruit drying, Zeng et al. found that a higher retention rate of total phenolic content (TPC), total flavonoid content (TFC), and vitamin content (VC) can be obtained in a certain range of infrared temperatures, but it will accelerate the Maillard reaction in the drying process of kiwifruit, resulting in serious color browning [9]. Huang et al. found that far-infrared drying increased the number of surface and internal micropores of stevia and improved the quality of dried products [10]. In the study of Cistanche, Jiang et al. found that far-infrared dried samples had more pores and better boundary integrity than did those subjected to natural drying [11]. Since food drying is mainly controlled by internal diffusion, internal resistance prevents water from being removed and diffused to the surface [12]. In order to increase the drying rate, it is necessary to use a pretreatment method to reduce the internal resistance as much as possible, reduce the initial water content of the raw material, or change the organizational structure of the raw material to shorten the drying time [13]. Ultrasonic waves (US), as a non-thermal radiation technology, interacts with the medium to produce mechanical effects, cavitation effects and thermal effects [14], which can change the organizational structure, reduce the resistance of water migration in the material, and promote internal water diffusion [15]. Ultrasonic pretreatment can effectively enhance heat transfer, improve drying efficiency, and inhibit the deterioration of material quality. Studies have shown that ultrasonic pretreatment, combined with far-infrared drying, can better retain the chemical components, particularly antioxidant activity and flavonoid content, of saffron [16]. In the study of *Lycium barbarum*, Zhang et al. found that the use of ultrasound can improve the quality of far-infrared dried *Lycium barbarum* products, increase the micropores on the surface of the material, increase the capillaries, reduce the mass transfer resistance, and facilitate the diffusion of water in the material [17]. Xi et al. used ultrasound, combined with far-infrared drying, to dry potato slices, which significantly improved the porous structure of potatoes, enhanced the heat and mass transfer process, and helped to retain the TPC and TFC content [18].

In this study, we intend to investigate the effects of different ultrasonic action times, ultrasonic power, and ultrasonic frequency on the drying characteristics, active ingredients, and microstructure of licorice slices subjected to far-infrared drying, with a view to providing theoretical references for the industrial processing of licorice.

## 2. Materials and Methods

### 2.1. Test Materials and Reagents

The licorice used in the experiment was purchased from the licorice planting base in Gongjing Township, Yuzhong County, Gansu Province. In order to prevent the water loss of fresh licorice after excavation, after purchase, it was placed in a 4 °C constant temperature fresh-keeping cabinet. The test materials were selected to reflect a uniform appearance and size in the samples. The average moisture content of fresh licorice held at 105 °C for 24 h, as determined by the official AOAC method, was 52.15% [19].

### 2.2. Chemicals

Liquiritin apioside (standard), glycyrrhizic acid (standard), isoliquiritin apioside (standard), neoisoliquiritin (standard), liquiritigenin (standard), retrochalcone (standard), liquiritin (standard), isoliquiritin (standard), phenol, 1,1-diphenyl-2-trinitrophenylhydrazine (DPPH), catechin, ascorbic acid, and forinol were used. The rest of the reagents, such as methanol, were analytically pure. All reagents were purchased from Chengdu Desite Biotechnology Co., Ltd., Chengdu, China.

### 2.3. Ultrasonic Pretreatment

Licorice samples of uniform size and water content were selected as test materials. The samples were cleaned, placed in distilled water, and pretreated by an ultrasonic device (KQ-300VDE three-frequency CNC ultrasonic cleaner, Kunshan Ultrasonic Instrument Co., Ltd., Shanghai, China). Based on the preliminary testing, the ultrasonic power (40 W, 60 W, 80 W), ultrasonic frequency (20 kHz, 40 kHz, 60 kHz), and ultrasonic treatment time (20 min, 30 min, 40 min) were selected as the adjustment parameters. After the sample pretreatment was completed, the sample was removed, and the excess water on the surface of the sample was absorbed with hygroscopic paper. The arrangement of the ultrasonic one-way test for licorice slices is shown in Table 1.

### 2.4. Drying Test Method

**Figure 1:**
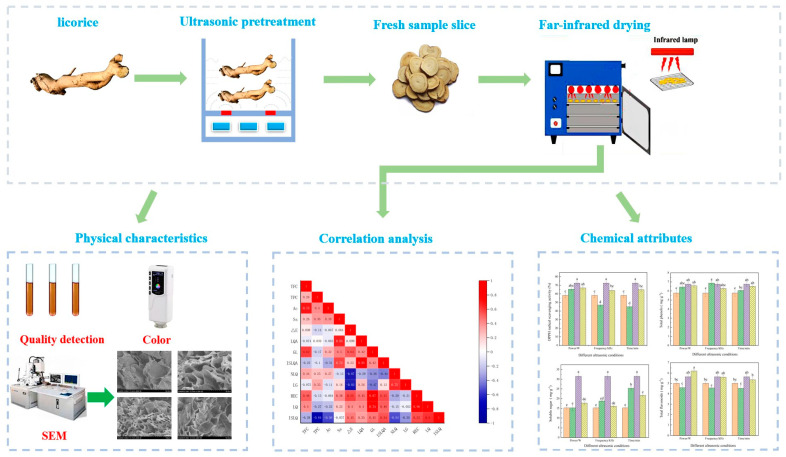
Schematic diagram of far-infrared drying and quality detection of licorice under different ultrasonic pretreatment conditions. The vertical bars indicate the standard deviation from the mean. The letters reveal significant differences (*p* < 0.05) according to the Duncan test.

The drying test was carried out in a far-infrared drying oven. Before the test, the equipment was adjusted to the preset parameters (drying power 900 W, temperature 50 °C) for preheating. After ultrasonic pretreatment, the samples were drained from the surface, sliced, weighed (120.0 ± 0.5) in a single layer, placed on trays, and dried in the far-infrared drying equipment (the optimal process for the far-infrared drying of licorice was obtained from the pre-test at a drying temperature of 50 °C and a slice thickness of 4 mm, so these were set as the fixed drying parameters). During the drying process, the sample trays were removed every 20 min, weighed on a digital balance with an accuracy of ±0.001 g (AUW-220D, Shimadzu, Japan), and then placed back in the drying oven to continue drying until the moisture content of the sample decreased by less than 12% [1]. Each group of tests was repeated three times, and the average value was taken as the test value. To verify the result of pretreatment on the of far-infrared drying effect on licorice, far-infrared dried licorice without ultrasonic pretreatment was selected as a control group for comparison. The experimental procedure is shown in Figure 1.

### 2.5. Calculation of Drying Parameters

#### 2.5.1. Moisture Content of the Dry Base

The formula for calculating the moisture content of the dry base is as follows [20].

(1) $$M_{t}=\frac{\left(M_{a}-M_{0}\right)}{M_{0}}\times 100\%$$

where *M_t_* is the dry basis moisture content (g · g^−1^), *M_a_* is the mass of the licorice slice at time *t* (g), and *M*_0_ is the mass of the dry matter of the licorice slice (g).

#### 2.5.2. Moisture Ratio

The moisture ratio of the licorice slices under different drying conditions was calculated by the following equation [20].

(2) $$MR=\frac{M_{t}}{M_{0}}$$

where *MR* is the moisture ratio of the licorice slices, *M_t_* is the dry basis moisture content of the licorice slices at moment *t* (g · g^−1^), and *M*_0_ is the initial dry basis moisture content of the licorice slices (g · g^−1^).

#### 2.5.3. Drying Rate

The drying rate of licorice slices was calculated using the following equation [21].

(3) $$DR=\frac{M_{t1}-M_{t2}}{t_{2}-t_{1}}$$

where *t*_1_ and *t*_2_ are arbitrary drying times, and *M_t_*_1_ and *M_t_*_2_ are the dry basis moisture content (g · g^−1^) of the licorice slices at moments *t*_1_ and *t*_2_, respectively.

### 2.6. Determination of Quality Indicators

#### 2.6.1. Preparation of Sample Extracts

The licorice slices were first crushed through an 80 mesh sieve [22]. A total of 1.0 g of powder was precisely weighted and placed it in a 25 mL 80% absolute ethanol stopper flask, rotated, vibrated (120 r/min) for 48 h under shaded room temperature, and then centrifuged for 10 min (rotation speed: 5000 r/min). The supernatant was diluted with absolute ethanol to 25 mL and stored in a refrigerator at 4 °C until subsequent determination and analysis of total phenolic content, antioxidant activity, soluble sugars, and total flavonoids [23]. Each test was repeated three times, and the average was then taken.

#### 2.6.2. Determination of Total Phenolic Content

The total phenol content was determined by the Folin–Ciocalteu method [24,25]. A total of 50 μL of extract solution was added to 2.0 mL of 10% Folin-Ciocalteu solution and 1.0 mL of 7.5% sodium carbonate solution, mixed and shaken for 5 min, and placed in a water bath at 37 °C for 1 h. The absorbance (A) was measured at 765 nm with a spectrophotometer, with the addition of no extract solution as a blank control. The standard curve of total phenol content was customized with gallic acid as the reference standard. The calculation formula of total phenol content is:

(4) $$TPC=\frac{V_{2}C_{1}}{V_{1}M}$$

where *C*_1_ represents the mass concentration of gallic acid, mg·g^−1^; *V*_1_ represents the volume of the sample extract, mL; *V*_2_ represents the total volume of the sample extract, mL; and *M* represents sample mass, g.

#### 2.6.3. Determination of Antioxidant Activity

The total antioxidant activity of organic active substances was determined using the DPPH method [26]. A total of 300 μL of extract solution was added to 3.0 mL of 0.1 mmol/L DPPH methanol solution under shaking at room temperature in darkness for 30 min, followed by the determination of the absorbance (*A*) value at 515 nm. According to the above operation, 75% ethanol was used as a blank control, and 500 μmol/L 90% ascorbic acid (ASA) methanol solution was used as a positive control. The antioxidant calculation formula is as follows:

(5) $$\mathrm{Inhibition}\;\mathrm{rate}=\frac{A_{0}-A}{A_{0}}\times 100\%$$

where *A* is the absorbance value of the sample solution, and *A*_0_ is the absorbance of the solution without sample.

#### 2.6.4. Determination of Soluble Sugar

The soluble sugar content was measured using the anthrone sulfuric acid method [27]. To 120 μL of the extract, 1.0 mL of 9% phenol solution was added and mixed thoroughly; 3.0 mL of concentrated sulfuric acid was subsequently added and the mixture was shaken for 5 min, reacted at room temperature for 30 min, and the A value at 485 nm was then measured. The standard curve of soluble sugar content was customized with glucose as a reference substance. The soluble sugar content calculation formula is:

(6) $$\mathrm{Soluble}\;\mathrm{sugars}=\frac{V_{2}C_{2}}{V_{1}M}$$

where *C*_2_ represents the mass concentration of soluble sugar, g · g^−1^; *V*_1_ represents the volume of the sample extract, mL; *V*_2_ represents the total volume of the sample extract, mL; and *M* represents sample mass, g.

#### 2.6.5. Determination of Total Flavonoids

The content of total flavonoids was determined using NaNO_2_-Al(NO_2_)_3_-NaOH [28]. To 800 μL of the extract, 2.0 mL of distilled water and 0.3 mL of 5% sodium nitrate solution were added, the mixture was shaken for 5 min, 0.3 mL of 10% aluminum chloride solution was added, the mixture was mixed and shaken for 5 min, and finally, 2.0 mL of 1 mol/L sodium hydroxide solution was added. The sample was allowed to fully react, using no extract as blank control, and the absorbance (A) value was measured at 510 nm. The standard curve of total flavonoids content was customized with catechin as reference substance. The total flavonoid content calculation formula is:

(7) $$TFC=\frac{V_{2}C_{3}}{V_{1}M}$$

where *C*_3_ represents the mass concentration of catechin, mg · g^−1^; *V*_1_ represents the volume of sample extract used, mL; *V*_2_ represents the total volume of sample extract, mL; and *M* represents the mass of sample, g.

#### 2.6.6. Color and Luster

The color of fresh licorice slices and licorice slices under different drying conditions were measured using a colorimeter (CR-10 type, Konica Minolta Ltd., Tokyo, Japan). The difference in color between the samples was represented by ∆*E*. The smaller the ∆*E*, the better the quality of the dried product. ∆*E* is calculated as follows:

(8) $$△E=\sqrt{{\left(L^{*}-L_{0}\right)}^{2}+{\left(a^{*}-a_{0}\right)}^{2}+{\left(b^{*}-b_{0}\right)}^{2}}$$

where L*, a*, and b* are the brightness, red-green value, and blue-yellow value of the dried licorice product. *L*_0_, *a*_0_, and *b*_0_ are the brightness, red-green value, and blue-yellow value of the fresh sample.

#### 2.6.7. Microstructure

The surface microstructure of the licorice slices under different drying conditions was observed using electron microscopy [29]. Before the test, each sample was cut into 5 mm × 5 mm pieces and then immediately fixed with 2.5% glutaraldehyde solution to stabilize the structure and composition of the biological system. After spraying, the samples were observed by SEM (magnification of ×300), and the acceleration voltage was 5.0 KV.

### 2.7. Determination of Bioactive Constituent Content in Licorice

Preparation of reference substances: a total of 3 mg of liquiritin apioside (LA), glycyrrhizic acid (GA), isoliquiritin apioside (ILA), neoisoliquiritin (NIL), liquiritigenin (LG), retrochalcone (RC), liquiritin (LQ), and isoliquiritin (ILQ) standards were accurately weighed to prepare a reference substance with a mass concentration of 1 mg·mL^−1^, which was then diluted into different concentration gradients for linear relationship investigation.

Preparation of test samples: The sample powder was sieved through an 80 mesh sieve, and 0.5 g was accurately weighed and placed in a 25 mL 80% anhydrous ethanol stopper triangular flask for ultrasonic treatment for 25 min (power 100 W, frequency 40 kHz). The constant volume with anhydrous ethanol was 25 mL, which was then centrifuged for 10 min, and the supernatant was filtered using a 0.22 μm filter membrane and injected for analysis.

The test materials used are as follows—chromatographic column: Agilent SB-C18 (250 mm × 4.6 mm, 5 μm); mobile phase: acetonitrile (A) −1% aqueous acetic acid (B); gradient elution program: 0–4 min (15–40% A), 4–8 min (40–65% A), 8–10 min (65–85% A), 10–12 min (85–15% A), 12–16 min (15–15% A); mobile phase flow rate: 1.0 mL/min; column temperature: 40 ℃; sample volume: 2 μL; and detection wavelength: 250 nm.

### 2.8. Statistical Analysis

Each group of tests was repeated three times and the average value was acquired. The obtained data were evaluated for moisture ratio and drying rate using Excel 2019. The curve and histogram were drawn by Origin 8.0. SPSS 24.0 was used for the analysis of variance (ANOVA). Tukey’s multiple-range test was used to analyze the significance of the mean difference, and the significance level was 0.05.

## 3. Results and Analysis

### 3.1. Effects of Drying Characteristics

**Figure 2:**
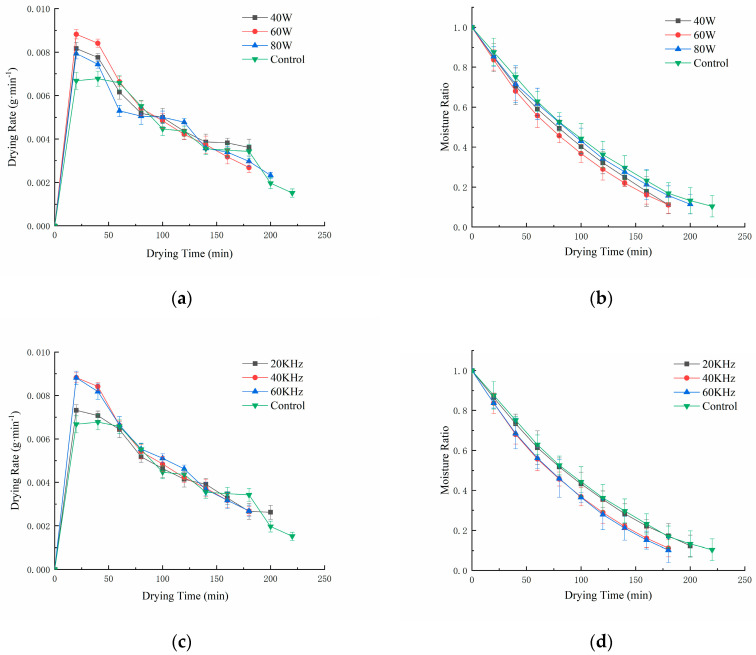

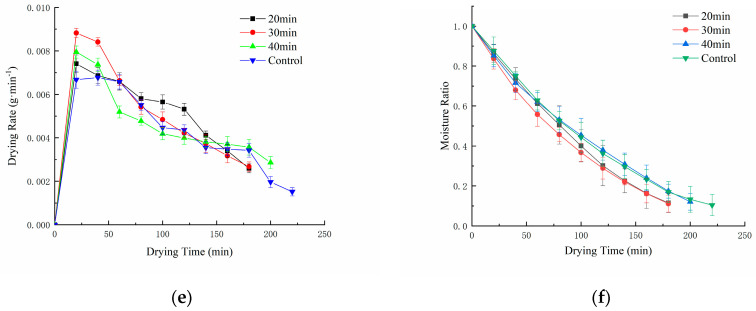
Drying rate curve (**a**) and moisture ratio curve (**b**) of licorice slices subjected to different ultrasonic power; drying rate curve (**c**) and moisture ratio curve (**d**) of licorice slices subjected to different ultrasonic frequency; drying rate curve (**e**) and moisture ratio curve (**f**) of licorice slices subjected to different ultrasonic time.

The effects of different ultrasonic powers on the moisture ratio and drying rate of licorice slices at an ultrasonic frequency of 40 kHz and an ultrasonic treatment time of 30 min are shown in Figure 2a,b. It can be seen from the figure that the drying time required for the moisture content to reach the safe level was shortened from 220 min to 180 min, saving 9.1–18.2%. It is worth noting that the drying rate curve of ultrasonic power 80 W is lower than that of 40 W and 60 W, and the drying time is the longest (200 min). This may be because the higher the ultrasonic power, the more water infiltrated inside the licorice [30]. Water can fill the intercellular space or enter the cells. The water in the intercellular space can be easily removed during the drying process. However, water that accumulates within the cells may hinder the drying process, as it must first diffuse through the cell membrane and then reach the outer layer of the sample in order to be vaporized [31]. It follows that sonication pretreatment at the optimal power allows the licorice sections to reach drying standards more quickly.

The effects of different ultrasonic frequencies on the moisture ratio and drying rate at 60 W ultrasonic power and 30 min ultrasonic treatment time are shown in Figure 2c,d. With increasing ultrasound frequency, the time required to dry licorice to concentrations below the safe moisture content level was 200, 180, and 180 min, respectively. Compared with the control group, the moisture ratio curve of licorice slices showed a greater decrease after pretreatment, and the drying time was shortened by 9.1%, 18.2%, and 18.2%, respectively. The higher the ultrasound frequency, the less time is required for drying, which is attributed to the “sponge effect” caused by ultrasound [32]. Under the high-frequency vibration of ultrasound, the internal structure of the licorice slices rapidly compresses and expands, generating a large number of microbubbles, which instantly generate strong kinetic and compression energy when they rupture. These energies can reduce the adhesion of water molecules tightly bound to the microtubule wall, thereby enhancing the fluidity of water and making it easier for water molecules to diffuse in licorice tablets. This energy will also change the structure of the liquid in licorice, as well as the force between water molecules, thereby promoting the internal diffusion rate of water. In addition, the higher the ultrasonic frequency, the stronger the perturbation effect generated by the ultrasonic wave, which is more conducive to enhancing the turbulence of water in the material inside the infrared radiation heat source area, increasing the degree of freedom of the hydrogen protons, improving the fluidity of water molecules, reducing the thickness and concentration difference of the solid–liquid mass transfer boundary, making it conducive to heat and mass transfer. Other studies have also shown that the “sponge effect” caused by ultrasound is beneficial to the absorption of infrared energy and the loss of water inside the material [33].

The effects of different ultrasonic treatment times on the moisture ratio and drying rate of licorice slices at an ultrasonic power of 60 W and an ultrasonic frequency of 40 kHz are shown in Figure 2e,f. It can be seen from the figure that the different ultrasonic treatment times affect the strengthening effect during the sample drying process. When the ultrasonic treatment was 30 min, the maximum drying speed and the minimum drying time could be achieved, but with the extension of the ultrasonic treatment time, the strengthening effect was no longer significant. This may occur because in the process of ultrasonic pretreatment, the osmotic exchange between the licorice slices and the ultrasonic medium is achieved by water loss and solids increase [34]. With the increase in ultrasonic treatment time, the solids content will increase accordingly. The cavitation effect of the ultrasonic waves will show a larger characteristic impedance in the solid, and its attenuation coefficient will be larger, which means that longer ultrasonic treatment times will weaken the strengthening effect [35]. Therefore, the more suitable range of ultrasonic treatment time is 20~30 min for the ultrasonic pretreatment of licorice prior to far-infrared drying.

The drying rate curve was analyzed. Under different drying conditions, the drying rate of each sample reached the maximum at about 20 min, and then decreased continuously. On the one hand, this is because the moisture content of the material is continuously reduced, which greatly increases the diffusion resistance of water in the sample; on the other hand, the attenuation coefficient of ultrasonic wave propagation in the material will increase with the decrease in the dry basis moisture content of the material, the mechanical effect and cavitation effect will be weakened, and the ultrasonic strengthening effect is not obvious [35].

### 3.2. Influence of Quality

#### 3.2.1. Influence of Total Flavonoid Content

**Figure 3:**
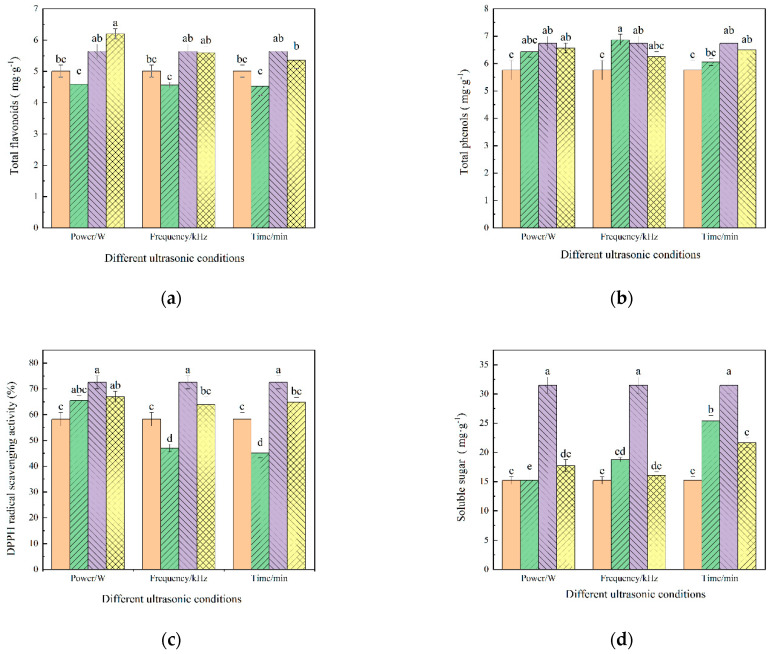
(**a**) Total flavonoids content; (**b**) total phenolic content; (**c**) DPPH radical scavenging activity; (**d**) Soluble sugar content. The total phenolic, antioxidant, soluble sugar, and total flavonoid content in licorice under different ultrasonic conditions. Control group (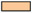), 40 W (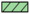), 60 W (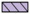), 80 W (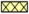). The vertical bars indicate the standard deviation from the mean. The letters reveal significant differences (*p* < 0.05) according to the Duncan test.

The effect of different ultrasonic pretreatment conditions on the TFC of licorice slices is shown in Figure 3a. It can be seen from the figure that with the increase in ultrasonic power, the retention of flavonoids in licorice slices increased gradually. When the ultrasonic power was 80 W, the content of flavonoids was the highest (1.984 mg catechin equivalent/g), at 23.4% higher than that of the control group (1.608 mg catechin equivalent/g), which was basically consistent with the results obtained in the study of sweet potatoes by Muhammad Tayyyab Rashid et al. [36]. This may be because with the enhancement of ultrasonic action, the drying time of the material is shortened, which reduces the oxidative decomposition of flavonoids. In addition, the high-intensity ultrasonic action makes the pores inside the licorice more porous [37], and it is easier to penetrate the interior of the cell to break the covalent bond between the polymers during far-infrared radiation, which is helpful for the synthesis of flavonoids [38]. With the extension of ultrasonic time, the flavonoid content first increased and then decreased, indicating that an extended ultrasonic treatment time was not conducive to the synthesis of flavonoids. This may be because the ultrasonic action itself will produce a thermal effect. With the increase in ultrasonic treatment time, the temperature in licorice is also rising, but at higher temperatures, the related flavonoid synthase will undergo structural and activity changes, resulting in some flavonoid synthase cannot be synthesized [10]. The previous study of this subject showed that under this condition, the drying process was relatively long, and the oxidative degradation of flavonoids may exist during the drying process, so that the content of flavonoids was lower than that without ultrasonic treatment.

#### 3.2.2. Effect of Total Phenolic Content

The effects of different ultrasonic pretreatment conditions on the TPC of licorice slices are shown in Figure 3b. It can be seen that the total phenol content of the samples after ultrasonic treatment was higher than that of the control group, and with the increase in ultrasonic power and the extension of ultrasonic treatment time, the total phenol content in licorice samples first increased and then decreased, and with the increase in ultrasonic frequency, the total phenol content decreased. The main reason may be that the shock wave and high-speed jet generated by ultrasonic cavitation damaged the cell wall of the licorice, promoting the release of polyphenols (such as chlorogenic acid, caffeic acid, rutin, and gallic acid) in the sample and increased the content of polyphenols in the material [39]. Moreover, ultrasonic treatment can reduce the content of dissolved oxygen in the licorice slices and slow down the oxidative decomposition of phenols. Nevertheless, if the ultrasonic power, ultrasonic frequency, and ultrasonic treatment time are too high, the degradation rate of phenolic substances such as hydroxybenzoic acid will increase. The study of Joanna Kroehnke et al. also confirmed this. They found that the content of phenolic compounds in carrots was significantly higher after ultrasound pretreatment compared to that of the unsonicated samples [40]. Observing the overall trend, it was found that the highest total phenolic content (0.686 mg/g) was obtained when the sonication power was 60 W, the sonication frequency was 20 kHz, and the sonication time was 30 min, which increased by 19.4% relative to the control group (0.576 mg gallic acid equivalent/g). This is because the mechanical action of ultrasound breaks the bond between the macromolecules and the phenolic compounds, and the phenolic compounds are released [41]. However, an ultrasonic frequency that is too high will destroy the integrity of the cells and cause oxidative decomposition. On the whole, high ultrasonic power, ultrasonic frequency, and ultrasonic treatment time are not conducive to the retention of total phenolic content.

#### 3.2.3. Effect of Antioxidant Capacity (DPPH)

The effects of different ultrasonic conditions on the antioxidant activity of licorice slices are shown in Figure 3c. It can be seen that the highest antioxidant capacity of the material (72.54%) was achieved when the sonication pretreatment conditions were 60 W, 40 kHz, and 30 min of sonication time, which was 24.6% higher than that of the control group (58.22%). With the increase in ultrasonic power, the antioxidant activity of the material showed a trend of first increasing and then decreasing, and its change rule was basically consistent with that of the phenols. Studies have shown that polyphenol content is an important factor affecting antioxidant activity because it can affect the oxidation stability of substances [36], and the addition of a second hydroxyl group to the aromatic ring adjacent or opposite to the phenolic compound can enhance the antioxidant capacity of materials [42]. After ultrasonic pretreatment, the antioxidant capacity of the licorice slices was better than that of the control because ultrasonic pretreatment could alleviate the loss rate of phenolic substances and enhance the antioxidant capacity of the samples [22]. However, ultrasound, due to its high temperature and high pressure, will generate a large number of free groups and initiate an oxidation reaction, thus reducing the antioxidant activity of the body.

#### 3.2.4. Effect of Soluble Sugars

The effects of different ultrasonic conditions on the soluble sugar content of licorice slices are shown in Figure 3d. It can be seen that the content of soluble sugars was consistently higher than that of the control under all conditions, except for the ultrasound power of 40 W, the ultrasound frequency of 40 kHz, and the ultrasound treatment time of 30 min, under which the content (15.225 mg glucose equivalent/g) was comparable to that of the control. Ultrasonic pretreatment contributes to the retention of soluble sugar, which may be due to the mechanical effect produced by the shearing force generated by ultrasound, which enhances the penetration of the solvent into the sample matrix, increases the contact surface area between the solid and liquid phases, and improves the diffusion rate of the solvent such that the soluble sugar content increases. The soluble sugar content tended to increase and then decrease with increasing ultrasonic power, ultrasonic frequency, and ultrasonic treatment time. The highest soluble sugar content (31.490 mg glucose equivalent/g) was found in the samples at a treatment time of 30 min at 60 W ultrasonic power and 40 kHz ultrasonic frequency, which was a two-fold increase relative to the control group (15.207 mg glucose equivalent/g). This is due to the enhanced mechanical and cavitation effects of ultrasound, which reduce the mass transfer resistance, facilitate the diffusion of water, shorten the time required for drying, and reduce the oxidation of soluble sugars [43]. The Maillard reaction may occur between protein and polysaccharide, which is a typical chemical reaction. It can produce some products with a large molecular weight and a medium molecular weight. These products will change the structure of polysaccharide, thus reducing its content. However, excessive ultrasonic intensity or higher temperatures will affect the occurrence of this reaction [44].

### 3.3. Effect of Ultrasonic Treatment on Bioactive Constituents Content

The effect of the ultrasonic pretreatment of far-infrared radiation drying on the content of active ingredients in licorice is shown in Table 2. The influence of different ultrasonic conditions on the effective components is quite different. For example, at a sonication time of 40 min, GA decreased by 12.8%, and NIL and LG increased by 49.3% and 50%, respectively, compared to the control group, while the content of the other active ingredients did not change significantly. This may be because glycyrrhetinic acid is less stable under heat, and after prolonged heating, the ether glycoside bond breaks and glucuronide is removed to produce glycyrrhetinic acid and glycyrrhetinic acid 3-O-glucuronide [45,46]. When the ultrasonic power increased from 40 W to 80 W, the contents of GA, NIL, RC, LQ, and ILQ increased by 24.03%, 16.35%, 60.56%, 61.76%, and 16.59%, respectively, while the contents of LA and ILA increased first and then decreased, and the maximum contents were 11.403 mg/g and 2.588 mg/g at 60 W, respectively. According to the literature, LA and ILA are flavonoids, and flavonoids mainly contain 2-phenylchromanone as the basic parent nucleus, and -OH is the main reactive group. It is presumed that the 7′-OH contained in them can be oxidized to a quinone structure under high temperatures. The high ultrasonic power is more likely to destroy their molecular structures [47]. The effects of different ultrasonic frequencies on the active components were observed. With the increase in ultrasonic frequency, the content of LG decreased, the content of LA, ILA, and NIL increased first and then decreased, and the content of GA, RC, LQ, and ILQ increased gradually.

### 3.4. Color and Luster

Color difference (∆*E*) is an important parameter for assessing the quality of dried samples [48]. From Table 3, it can be seen that the L*, a*, and b* values of fresh licorice were 38.95, 39.30, and 25.63, respectively. In the case of ultrasonic pretreatment, the b* values of the dried licorice slices were all smaller than those of the fresh samples, indicating that ultrasonic treatment affects the yellowness values of licorice. The a* values increased with increasing ultrasonic time, suggesting that the length of ultrasonic action had a significant effect on the glycyrrhiza redness values, possibly because changes in a* can be linked to the formation of colored compounds [49], which undergo enzymatic and non-enzymatic browning reactions during treatment—the brown pigments are formed from colorless polyphenols and lead to changes in optical properties [50]. At the same time, longer ultrasonic action will increase the gap between cells, form more micropores, damage the surface of the material, and increase the effective area of contact with the ultrasonic medium, resulting in color changes. [51]. With the increase in ultrasonic frequency, the Δ*E* of licorice slices showed a declining trend, indicating that the higher the frequency, the better the color of licorice.

### 3.5. Correlation Analysis

**Figure 4:**
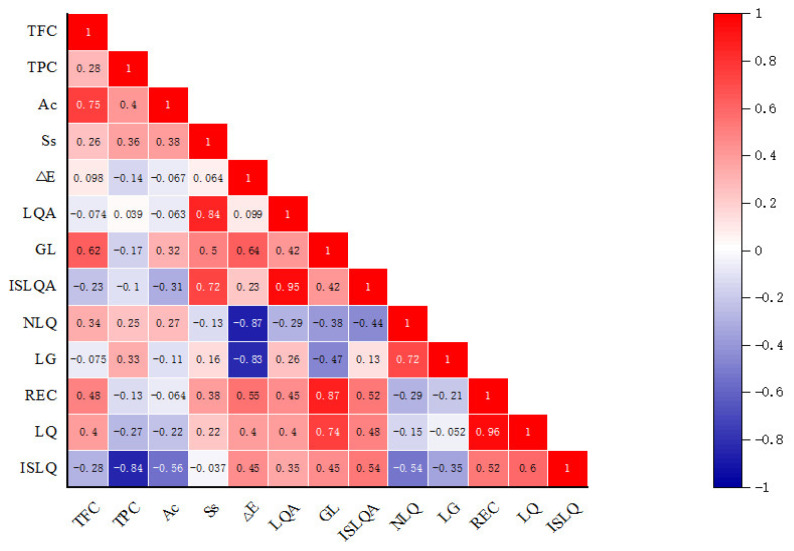
Correlation analysis between the indicators under different pretreatment conditions.

The correlation between the quality indexes of licorice slices is shown in the Figure 4. It can be seen that TFC is positively correlated with LQ, REC, NLQ, GL, ΔE, Ss, Ac, and TPC. The higher the TFC content, the greater the correlation with Ac (r = 0.75). Deng et al. also revealed that the antioxidant activity was positively correlated with TPC [19]. There was a significant positive correlation between REC and LQ (r = 0.96). TPC was negatively correlated with ISLQ (r = −0.84). Ss was positively correlated with LQA and ISLQA, and the correlation was significant (r = 0.84, r = 0.72). By observing the correlation between Δ*E* and each component, it was found that the index was negatively correlated with NLQ and LG, and positively correlated with other components. The positive correlation coefficient between LG and ISLQA was 0.95, and the correlation was extremely significant. On the whole, there is a significant correlation between pretreatment conditions and the quality indexes of licorice.

### 3.6. Microstructural Analysis

**Figure 5:**
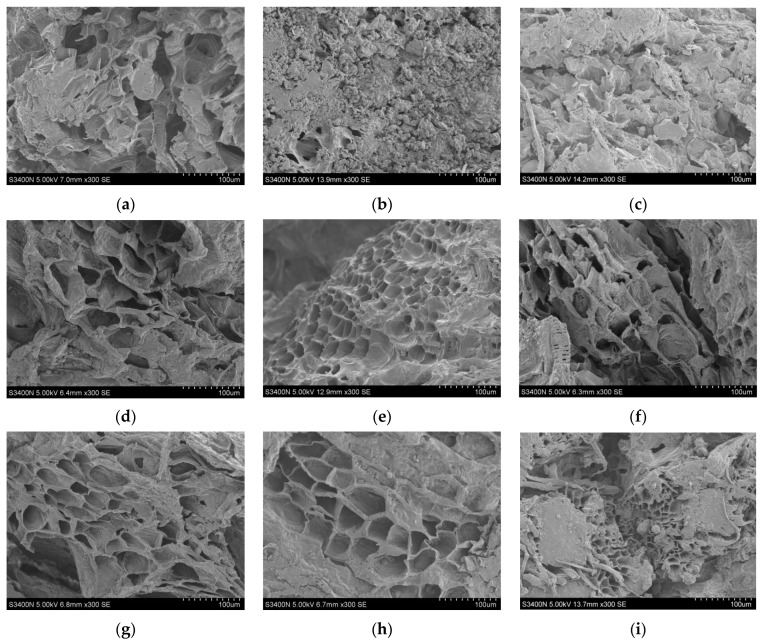
(**a**) Natural drying; (**b**) control; (**c**) 40 W, 40 kHz, 30 min; (**d**) 60 W, 40 kHz, 30 min; (**e**) 80 W, 40 kHz, 30 min; (**f**) 60 W, 20 kHz, 30 min; (**g**) 60 W, 60 kHz, 30 min; (**h**) 60 W, 40 kHz, 20 min; (**i**) 60 W, 40 kHz, 40 min. Microstructure of far infrared dried licorice under different pretreatment conditions.

In the drying process, the microstructure of the material has an important influence on the heat and mass transfer and water diffusion characteristics [49]. The microstructure of the dried products dried under different ultrasonic pretreatment conditions is shown in Figure 5. It can be clearly seen that the licorice slice tissue will continue to shrink during the drying process, and the cavity will become smaller, while the cell tissue structure of the dried products resulting from natural drying (Figure 5a) without ultrasonic pretreatment (Figure 5b) is more compact and denser. When the ultrasonic frequency was 40 kHz and the treatment time was 30 min, the higher the ultrasonic power, the clearer the arrangement of the cell structure microchannels on the surface of the licorice slices (Figure 5c–e). When the ultrasonic power was 60 W and the time was 30 min, regardless of the frequency, the microchannel of the cell structure on the surface of the licorice slices showed little change, indicating that the ultrasonic frequency had an insignificant effect. When the ultrasonic power was 60 W and the frequency was 40 kHz, with the increase in ultrasonic action time, the structure of the material cells and the formed microchannels was gradually destroyed, and the tissue integrity of the cells was also reduced (Figure 5h,d,i). In addition, when the ultrasonic pretreatment power was 80 W and the frequency was 40 kHz for 30 min, the skeletal structure of the licorice slice cells remained in good condition, and the microchannel was clearly visible. Under this condition, the fiber network structure could be observed.

## 4. Conclusions

In this study, far-infrared-dried licorice slices were pretreated using ultrasound technology, and the effects of different ultrasound conditions on drying characteristics, quality characteristics and microstructure were investigated. The results show that ultrasonic pretreatment mainly affects the microstructure changes, such as cell destruction and microchannel formation. When the ultrasonic pretreatment is carried out for a long period of time, the formation of the microchannel increases the porosity of the sample and enhances the subsequent drying process, but the ultrasonic effect will be weakened if the treatment time is excessive. In addition, the high frequency vibration is more favorable to the absorption of infrared energy and the loss of moisture within the material. Finally, it was confirmed that the extension of ultrasonic pretreatment time and excessive power would weaken the effect of ultrasonic pretreatment. The overall quality of the licorice slices was improved by ultrasonic pretreatment. The combination of ultrasonic power of 60 W, ultrasonic frequency of 40 kHz, and ultrasonic time of 30 min was found to be the best combination of pretreatment parameters. In addition, the retention of active ingredients in licorice is closely related to the integrity of its cell structure. By observing the microstructure, it was found that the surface cell structure changed to form microchannels, which provided more mass transfer paths for water migration. Therefore, ultrasonic pretreatment, combined with far-infrared drying, is an efficient method to obtain the retention of multiple components in licorice slices.

In this paper, the analysis of the drying characteristics and quality properties of licorice slices under ultrasonic pretreatment followed by far-infrared drying is investigated, but the more specific heat and mass transfer process inside the slices was still unclear. In future research, the heat and mass transfer mechanism of licorice slices should be studied in depth through simulation in order to provide a theoretical basis for the optimization of the drying process at a later stage.

## Figures and Tables

**Table 1:** Ultrasonic single-factor test of cistanche slices.

Experimental Number	Ultrasonic Power (W)	Ultrasonic Frequency (kHz)	Ultrasonic Treatment Time (min)
1	60	20	30
2	60	40	30
3	60	60	30
4	40	40	30
5	80	40	30
6	60	40	20
7	60	40	40

**Table 2:** Effective component content of licorice under different drying conditions (mg/g).

Different Ultrasound Conditions		LA	GA	ILA	NIL	LG	RC	LQ	LLQ
Control		7.96 ± 0.12 ^cd^	26.12 ± 1.50 ^ab^	2.10 ± 0.05 ^bc^	0.93 ± 0.14 ^a^	2.23 ± 0.18 ^bc^	1.89 ± 0.22 ^bc^	27.04 ± 0.27 ^c^	1.14 ± 0.19 ^d^
40 kHz 30 min	40 W	6.76 ± 0.18 ^d^	22.15 ± 1.05 ^d^	1.55 ± 0.12 ^d^	1.02 ± 0.20 ^a^	1.54 ± 0.05 ^e^	1.63 ± 0.11 ^c^	20.26 ± 0.75 ^d^	1.69 ± 0.14 ^cd^
	60 W	11.40 ± 1.02 ^ab^	27.25 ± 1.60 ^a^	2.59 ± 0.07 ^b^	1.02 ± 0.12 ^a^	2.08 ± 0.14 ^bcd^	2.41 ± 0.23 ^ab^	29.15 ± 0.37 ^b^	1.84 ± 0.11 ^c^
	80 W	6.80 ±0.25 ^d^	27.47 ± 1.05 ^a^	1.63 ± 0.11 ^cd^	1.18 ± 0.16 ^a^	1.75 ± 0.15 ^cde^	2.61 ± 0.13 ^a^	32.78 ± 0.91 ^a^	1.97 ± 0.13 ^c^
60 W 30 min	20 kHz	9.45 ± 0.13 ^b^	22.31 ± 0.42 ^cd^	2.34 ± 0.12 ^b^	1.02 ± 0.14 ^a^	2.57 ± 0.13 ^b^	2.19 ± 0.18 ^abc^	28.19 ± 0.63 ^bc^	1.66 ± 0.19 ^cd^
	40 kHz	11.40 ± 1.02 ^ab^	27.25 ± 1.60 ^a^	2.59 ± 0.07 ^b^	1.02 ± 0.12 ^a^	2.08 ± 0.14 ^bcd^	2.41 ± 0.23 ^ab^	29.15 ± 0.37 ^b^	1.84 ± 0.11 ^c^
	60 kHz	9.02 ± 0.33 ^b^	27.49 ± 0.54 ^a^	2.06 ± 0.19 ^bcd^	0.94 ± 0.14 ^a^	1.63 ± 0.21 ^de^	2.49 ± 0.09 ^ab^	31.90 ± 0.58 ^a^	3.03 ± 0.26 ^a^
60 W 40 kHz	20 min	12.15 ± 0.22 ^a^	27.25 ± 0.46 ^a^	3.19 ± 0.23 ^b^	0.90 ± 0.15 ^a^	1.89 ± 0.06 ^bcd^	2.63 ± 0.20 ^a^	33.66 ± 0.77 ^a^	4.29 ± 0.30 ^a^
	30 min	11.40 ± 1.02 ^ab^	27.25 ± 1.60 ^a^	2.59 ± 0.07 ^b^	1.02 ± 0.12 ^a^	2.08 ± 0.14 ^bcd^	2.41 ± 0.23 ^ab^	29.15 ± 0.37 ^b^	1.84 ± 0.11 ^c^
	40 min	9.77 ± 0.44 ^bc^	22.77 ± 1.65 ^cd^	2.08 ± 0.21 ^bcd^	1.39 ± 0.26 ^a^	3.35 ± 0.25 ^a^	2.02 ± 0.19 ^abc^	27.64 ± 0.83 ^bc^	1.65 ± 0.14 ^cd^

Note: Data are expressed as means ± standard deviation of triplicate samples. The letters in the same column reveal significant differences (*p* < 0.05) according to the Duncan test.

**Table 3:** Color value of far-infrared dried licorice slices under different pretreatment conditions.

Different Ultrasound Factors		L*	a*	b*	∆*E*
Fresh Sample		78.10 ± 0.03 ^b^	1.91 ± 0.32 ^a^	28.56 ± 0.90 ^a^	—
40 kHz 30 min	40 W	81.39 ± 0.54 ^a^	2.23 ± 0.08 ^a^	24.60 ± 1.17 ^c^	4.93 ± 0.42 ^c^
	60 W	75.71 ± 0.68 ^c^	1.79 ± 0.41 ^a^	25.00 ± 0.38 ^bc^	4.42 ± 0.67 ^c^
	80 W	77.73 ± 0.02 ^b^	2.18 ± 0.09 ^a^	23.45 ± 0.67 ^bc^	4.64 ± 0.82 ^c^
60 W 30 min	20 kHz	78.83 ± 0.17 ^b^	2.00 ± 0.81 ^a^	22.93 ± 0.73 ^bc^	5.28 ± 0.81 ^c^
	40 kHz	75.71 ± 0.68 ^c^	1.79 ± 0.41 ^a^	25.00 ± 0.38 ^bc^	4.42 ± 0.67 ^c^
	60 kHz	77.49 ± 0.76 ^b^	2.47 ± 0.08 ^a^	25.94 ± 1.79 ^abc^	3.97 ± 2.07 ^c^
60 W 40 kHz	20 min	63.89 ± 0.51 ^e^	0.06 ± 0.16 ^b^	24.74 ± 0.37 ^bc^	14.87 ± 0.69 ^a^
	30 min	75.71 ± 0.68 ^c^	1.79 ± 0.41 ^a^	25.00 ± 0.38 ^bc^	4.42 ± 0.67 ^c^
	40 min	68.35 ± 0.28 ^d^	2.07 ± 0.59 ^a^	26.27 ± 0.06 ^ab^	10.29 ± 0.14 ^b^

Note: Data are expressed as means ± standard deviation of triplicate samples. The letters in the same column reveal significant differences (*p* < 0.05) according to the Duncan test.

## Data Availability

The datasets generated for this study are available on request to the corresponding author.
